# Liver Protection of a Low-Polarity Fraction from *Ficus pandurata* Hance, Prepared by Supercritical CO_2_ Fluid Extraction, on CCl_4_-Induced Acute Liver Injury in Mice via Inhibiting Apoptosis and Ferroptosis Mediated by Strengthened Antioxidation

**DOI:** 10.3390/molecules28052078

**Published:** 2023-02-22

**Authors:** Weibo Dai, Xiaoyan Pang, Weiwen Peng, Xinyi Zhan, Chang Chen, Wenchang Zhao, Congyan Zeng, Quanxi Mei, Qilei Chen, Weihong Kuang, Zhanping Gou, Xianjing Hu

**Affiliations:** 1 Pharmacology Laboratory, Zhongshan Hospital, Guangzhou University of Chinese Medicine, Zhongshan 528401, China; 2 Guangdong Provincial Key Laboratory of Research and Development of Natural Drugs, And School of Pharmacy, Guangdong Medical University, Dongguan 523808, China; 3 Shenzhen Baoan Authentic TCM Therapy Hospital, Shenzhen 518101, China; 4 School of Chinese Medicine, Hong Kong Baptist University, Hong Kong 999077, China; 5 Dongguan Key Laboratory of Chronic Inflammatory Diseases, The First Dongguan Affiliated Hospital, Guangdong Medical University, Dongguan 523121, China

**Keywords:** *Ficus pandurata* Hance, liver injury, supercritical CO_2_ fluid extraction, apoptosis, ferroptosis, antioxidation

## Abstract

*Ficus pandurata* Hance (FPH) is a Chinese herbal medicine widely used for health care. This study was designed to investigate the alleviation efficacy of the low-polarity ingredients of FPH (FPHLP), prepared by supercritical CO_2_ fluid extraction technology, against CCl_4_-induced acute liver injury (ALI) in mice and uncover its underlying mechanism. The results showed that FPHLP had a good antioxidative effect determined by the DPPH free radical scavenging activity test and T-AOC assay. The in vivo study showed that FPHLP dose-dependently protected against liver damage via detection of ALT, AST, and LDH levels and changes in liver histopathology. The antioxidative stress properties of FPHLP suppressed ALI by increasing levels of GSH, Nrf2, HO-1, and Trx-1 and reducing levels of ROS and MDA and the expression of Keap1. FPHLP significantly reduced the level of Fe^2+^ and expression of TfR1, xCT/SLC7A11, and Bcl2, while increasing the expression of GPX4, FTH1, cleaved PARP, Bax, and cleaved caspase 3. The results demonstrated that FPHLP protected mouse liver from injury induced by CCl_4_ via suppression of apoptosis and ferroptosis. This study suggests that FPHLP can be used for liver damage protection in humans, which strongly supports its traditional use as a herbal medicine.

## 1. Introduction

According to a retrospective study in China, the annual incidence of liver injury is ~23 per 100,000, which inevitably leads to a substantial socioeconomic burden [1]. A series of factors, such as alcohol, drugs, viruses, and being overweight, can cause ALI (acute liver injury) [2]. The common symptoms of ALI include weakness, fatigue, nausea, swollen abdomen, itching, and jaundice [3], and the clinical symptoms include abnormal levels of alanine transaminase (ALT) and aspartate aminotransferase (AST), which increase the risk of liver fibrosis, cirrhosis, and even acute hepatic failure [4,5].

It was previously reported that the pathological mechanisms of liver injury involve cytochrome P450 (CYP450) metabolism disorder, inflammation, oxidative stress, and cell apoptosis [6]. The majority of drug metabolism is mediated by the CYP450 enzyme system in the liver, and oxidative damage induced by free radicals has become an important factor in hepatotoxicity. The mitochondria is the main organelle that produces oxidative metabolites and is also susceptible to ROS (reactive oxygen species) attack, which can lead to structural and functional disturbances, thereby inducing hepatocyte apoptosis [7]. Ferroptosis, a regulated form of cell death induced by iron-dependent lipid peroxidation, has become an important factor in many diseases [8]. Increasing evidence indicates that ferroptosis is also involved in the process of liver injury [9]. Under the depletion of glutathione (GSH) and inactivation of glutathione peroxidase 4 (GPX4), Fe^3+^ can be reduced to Fe^2+^ and iron overloading promotes lipid peroxidation and the generation of mitochondrial ROS [10]. The accumulated redox-active iron plays an important role in the Fenton reaction and the production of lipid peroxidation, as well as the disintegration of ferritin [11], which leads to ferroptosis [12]. Therefore, it is exceptionally critical to prevent liver injury by inhibiting hepatocyte apoptosis and ferroptosis via targeting of oxidative stress.

Currently, the therapeutic strategies for ALI mainly include gastric lavage, activated charcoal, ipecacuanha (an emetic) [13], hemodialysis [14], as well as pharmacotherapies (e.g., glutathione, glycyrrhizin, S-adenosylmethionine) [15]. Pharmacotherapy can only prevent further liver damage from gastric residues, but it cannot repair the damaged liver and may cause discomfort to patients [16]. The drugs used in the clinic can induce a series of side effects, such as hypertension, obesity, and insulin resistance [17]. N-acetyl-L-cysteine (NAC) is a commonly used drug for treating liver injury in the clinic but cannot be administered to children because it induces rash and nausea [18,19]. New drugs or alternative remedies are still largely in demand. Traditional ethnic herbal medicines have a long-history of application in humans and are beneficial for liver health due to their edible properties and few adverse effects [10]. Hence, developing new drugs from ethnic herbal medicines is a good approach for protecting the liver from injury.

*Ficus pandurata* Hance (FPH), a Chinese ethnic herbal medicine with medicine and food homology, has been widely utilized for liver health care in Southeast China. In addition, the “*Atlas of 100 kinds of well-chosen wild vegetables in Zhejiang*” also recorded that FPH possessed the functions of “regulating Qi”, i.e., activating blood circulation along with dispelling dampness and detoxication. In this study, the low-polarity fraction from *Ficus pandurata* Hance (FPHLP) was obtained by supercritical CO_2_ fluid extraction technology. Gas chromatography-mass spectrometry (GC-MS) was applied to analyze the chemical composition of FPHLP and the antioxidant capacity of FPHLP was evaluated by 2,2-diphenyl-1-picrylhydrazil (DPPH), total antioxidant capacity (T-AOC), and flow cytometry assays. The alleviation function of FPHLP against CCl_4_-induced ALI in mice was evaluated via biochemical assay and hematoxylin & eosin (H&E) staining, and its mechanism was systematically determined by enzyme-linked immunosorbent assay (ELISA), western blotting, and immunohistochemistry (IHC) assays.

## 2. Results

### 2.1. Antioxidation Effects of FPHLP 

**Figure 1:**
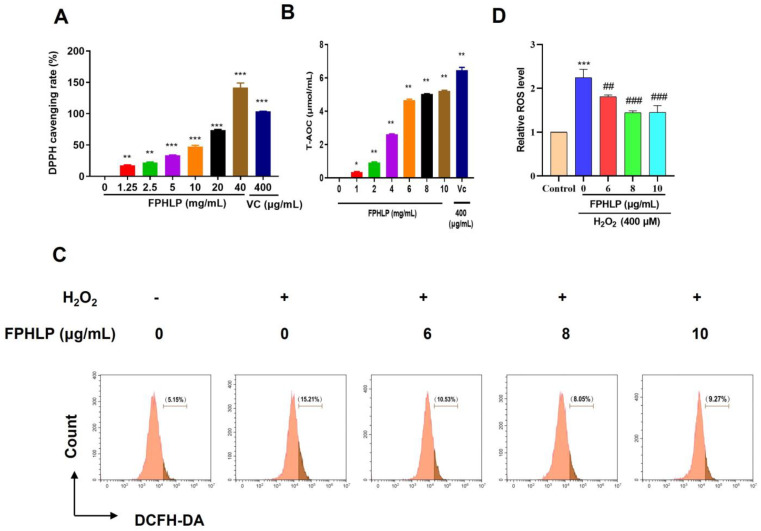
Antioxidation activity in vitro. (**A**) DPPH radical scavenging activity of FPHLP. (**B**) Total antioxidant activity of FPHLP. (**C**) Effect of FPHLP on H_2_O_2_-induced oxidative stress in HepG2 cells. Levels of ROS in HepG2 cells with or without FPHLP treatment (0, 6, 8, and 10 µg/mL) after H_2_O_2_ stimulation (0.4 mM). (**D**) Quantitative analysis of flow cytometry results. Results are expressed as the mean ± SEM (*n* = 3). In (**A**,**B**), vs. 0 mg/mL group, * *p* < 0.05, ** *p* < 0.01, *** *p* < 0.001; in (**C**), vs. control group, *** *p* < 0.001, vs. H_2_O_2_-induced group, ## *p* < 0.01, ### *p* < 0.001.

The antioxidation properties of FPHLP were evaluated by DPPH radical scavenging activity and T-AOC assays, as well as ROS detection via flow cytometry. As a result, FPHLP exhibited good DPPH radical scavenging activity in a concentration-dependent manner, with the highest clearance rate of 141.5% at a concentration of 40 mg/mL (Figure 1A). The T-AOC activity of FPHLP was measured according to the ferric reducing antioxidant power (FRAP) method, and the results showed that FPHLP exhibited good antioxidant activity in a dose-dependent manner (Figure 1B). The flow cytometry results also showed that the ROS level was increased in the H_2_O_2_-treated group, indicating that the model of H_2_O_2_-induced oxidative stress in HepG2 cells was successfully established. After FPHLP treatment (6, 8, and 10 µg/mL), the high levels of ROS induced by H_2_O_2_ were significantly reversed (Figure 1C,D). These results suggested that FPHLP had good antioxidative stress potency.

### 2.2. FPHLP Protects against CCl_4_-Induced Liver Injury

**Figure 2:**
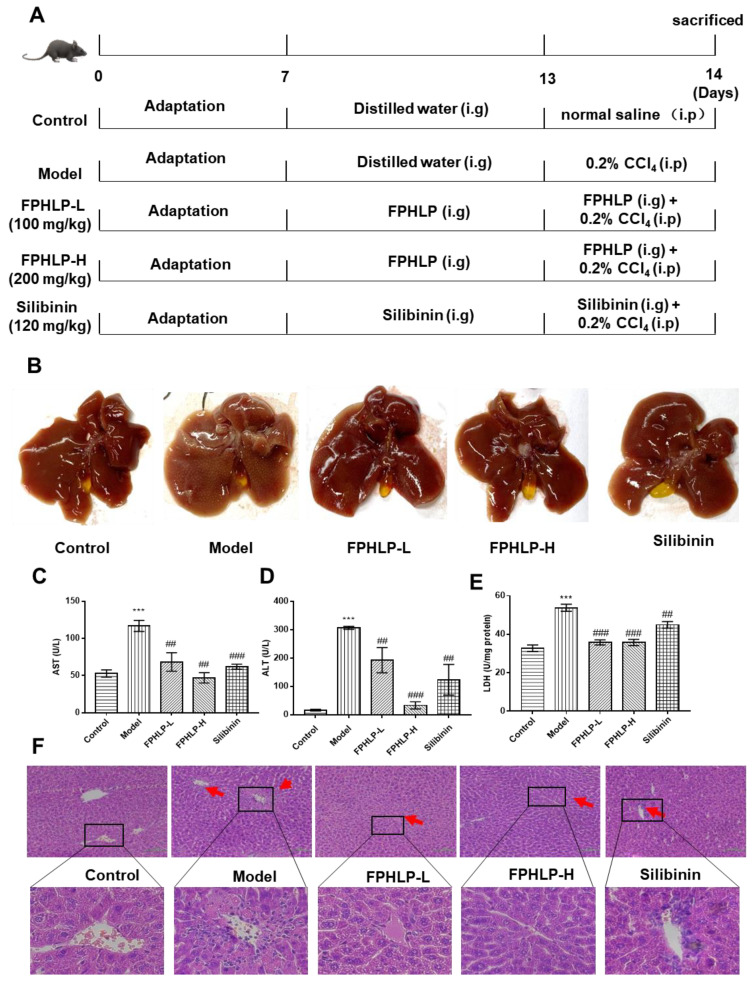
FPHLP protects the liver from damage in the ALI mouse model induced by CCl_4_. (**A**) Design of anti-ALI study in mouse model induced by CCl_4_. (**B**) Representative photographs of liver tissues. Effect of FPHLP on levels of AST (**C**) and ALT (**D**) in serum and LDH (**E**) in liver homogenate of ALI model mice. (**F**) Liver tissues were stained with H&E for histopathological analysis (original magnification, 200×; scale bar = 100 µm; the red arrows indicate the locations of positive cells for H&E analysis). Results are expressed as the mean ± SEM. vs. control group, *** *p* < 0.001; vs. CCl_4_-treated only group, ^##^ *p* < 0.01, ^###^ *p* < 0.001.

To explore the protective effects of FPHLP against liver injury, C57BL6/J mice were intraperitoneally injected with CCl_4_ to establish the acute liver injury (ALI) model and intragastrically administered FPHLP. As a result, the appearance of the liver tissue obviously changed after CCl_4_ induction; most of the hepatic lobules appeared yellow and granular lesions were observed. Meanwhile, the appearance of injury in the liver tissues of the FPHLP- and silibinin-treated groups was alleviated (Figure 2B), showing that FPHLP protected against ALI induced by CCl_4_. Furthermore, as shown in Figure 2C,D, the levels of ALT and AST in the serum of the model group were sharply enhanced after CCl_4_ induction and the level of LDH in the liver homogenate of the model group was significantly higher than that of the control group (Figure 2E), indicating that the liver injury mouse model was successfully established. Meanwhile, the ALT, AST, and LDH levels of the FPHLP- and silibinin-treated groups were significantly reversed, suggesting that FPHLP and silibinin had good protective effects against liver injury in mice. Additionally, the H&E staining assay showed that the liver tissues of the model group had typical features of liver injury, such as inflammatory infiltration and enlarged vacuoles. After FPHLP and silibinin treatments, the classical features of pathological changes in the liver tissues were remarkably ameliorated (Figure 2F). These data indicated that FPHLP could significantly alleviate the acute liver injury induced by CCl_4_ in mice.

### 2.3. FPHLP Protects Liver Injury via Inhibiting Apoptosis

**Figure 3:**
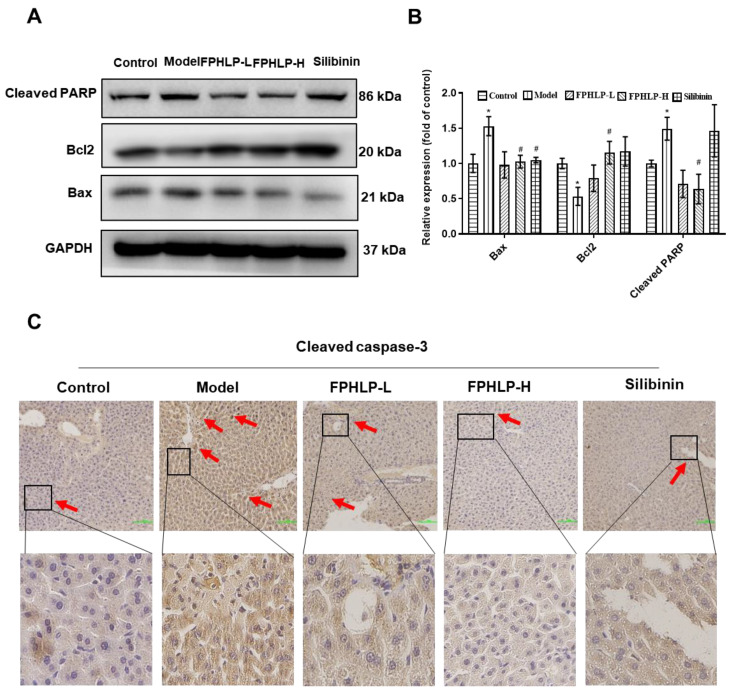
FPHLP protects the liver from damage in mice induced by CCl_4_ via inhibition of apoptosis. The expression of apoptosis proteins, including cleaved PARP, Bcl2, and Bax, in the liver tissues of ALI model mice were evaluated by western blotting (**A**) and quantitatively analyzed (*n* = 3) (**B**). (**C**) The expression of cleaved caspase-3 in the liver tissues of ALI model mice was detected by IHC (original magnification, 200×; scale bar = 100 µm; the red arrows indicate the locations of positive cells for IHC analysis). Results are expressed as the mean ± SEM. vs. control group, * *p* < 0.05; vs. CCl_4_-treated only group, ^#^ *p* < 0.05.

Liver injury can cause hepatocyte death, which is irreversible. Hence, the protective effect of FPHLP against hepatocyte apoptosis was evaluated via western blotting and IHC assays. The results showed that the expression of cleaved PARP and Bax in the liver tissues of mice was upregulated, while that of Bcl2 was reduced after CCl_4_ injection. However, after FPHLP treatment, the expression of cleaved PARP, Bax, and Bcl2 was significantly reversed, suggesting that FPHLP effectively suppressed hepatocyte apoptosis (Figure 3A,B). Meanwhile, the expression of cleaved caspase-3 in the liver tissues was detected via IHC assay, and the results showed that the expression of cleaved caspase-3 was increased in the CCl_4_-treated group compared to that in the control group, while that in the FPHLP-treated groups was sharply suppressed (Figure 3C). These results suggested that FPHLP effectively protected against apoptosis.

### 2.4. FPHLP Protects Liver Injury via Strengthening Antioxidative Activity

**Figure 4:**
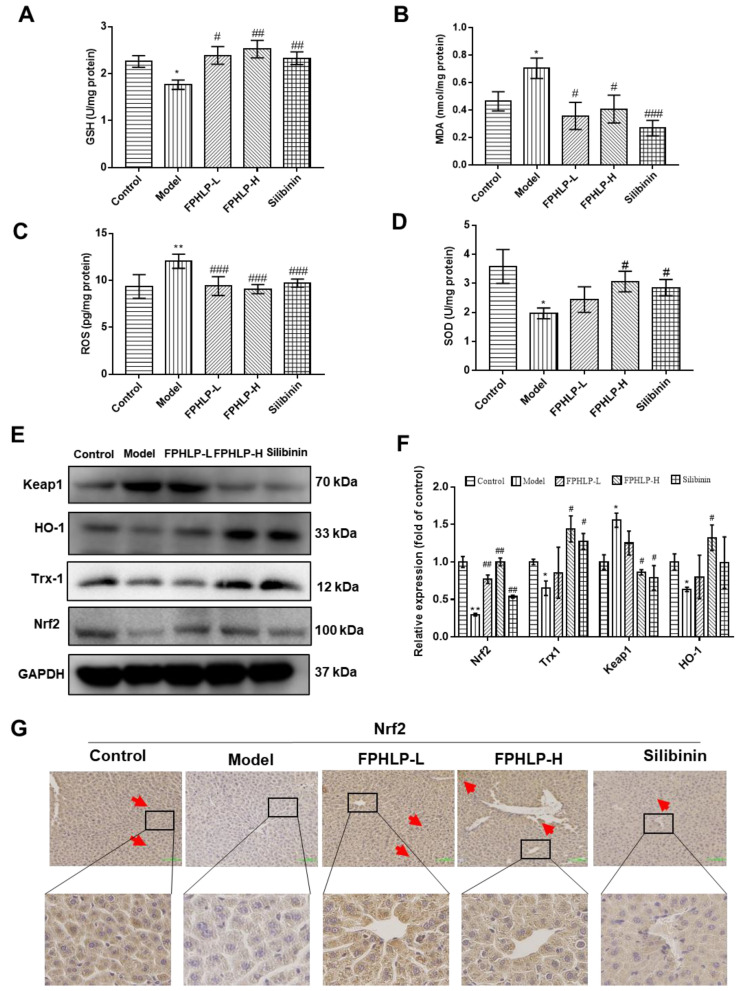
FPHLP alleviates liver injury in mice induced by CCl_4_ via strengthening antioxidative activity. Effect of FPHLP on levels of GSH (**A**), MDA (**B**), ROS (**C**), and SOD (**D**) in the liver homogenate detected by ELISA. (**E**) The expression of Keap1, HO-1, Trx-1, and Nrf2 in the liver tissues of ALI model mice was evaluated by western blotting and quantitatively analyzed (*n* = 3) (**F**). (**G**) The expression of Nrf2 in the liver tissues of ALI model mice was detected by IHC (original magnification, 200×; scale bar = 100 µm; the red arrows indicate the locations of positive cells for IHC analysis). Results are expressed as the mean ± SEM. vs. control group, * *p* < 0.05, ** *p* < 0.01; vs. CCl_4_-treated only group, ^#^ *p* < 0.05, ^##^ *p* < 0.01, ^###^ *p* < 0.001.

Oxidative stress has been recognized as an important mechanism underlying the pathophysiology of ALI [20]. In the present study, the influence of FPHLP on oxidative stress in the liver tissues of CCl_4_-induced ALI model mice was investigated via ELISA assays, western blotting, and IHC assays. As shown in Figure 4A–D, the levels of SOD and GSH, two important antioxidation parameters, were significantly decreased and the levels of ROS and MDA were significantly increased after CCl_4_ induction (*p* < 0.05 vs. control group), while those of the FPHLP-treated groups were significantly reversed (*p* < 0.05 vs. model group). Additionally, the effect of FPHLP on the expression of oxidative stress-related proteins, including Keap1, Nrf2, HO-1, and Trx-1, was assessed. The results showed that CCl_4_ markedly increased the expression of Keap1 and decreased the expression of Nrf2 and its downstream factor HO-1 as well as Trx-1, while FPHLP treatment strongly reversed these effects (Figure 4E,F), indicating that FPHLP effectively suppressed oxidative stress in the liver tissues. The IHC assay was also used to detect the expression of Nrf2, the critical factor of the Keap1/Nrf2/HO-1 pathway, in the liver tissues of ALI model mice, and the results showed that FPHLP enhanced the expression of Nrf2 in mice with CCl_4_-induced ALI (Figure 4G). Altogether, these data demonstrated that FPHLP strongly alleviated the acute liver injury in mice by enhancing the antioxidative capacity.

### 2.5. FPHLP Protects Liver Injury via Inhibiting Ferroptosis

**Figure 5:**
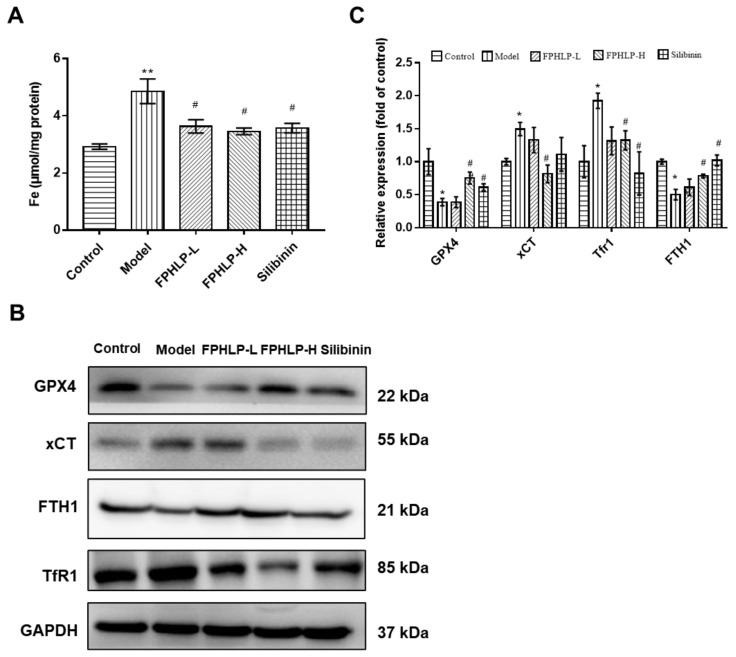
FPHLP protects the liver from damage in ALI model mice via inhibiting ferroptosis. (**A**) Effect of FPHLP on the level of Fe^2+^ in the liver homogenate of ALI model mice. Protein expression of GPX4, xCT, FTH1, and TfR1 in the liver tissues were determined by western blotting (**B**) and quantitatively analyzed (*n* = 3) (**C**). Results are expressed as the mean ± SEM. vs. control group, * *p* < 0.05, ** *p* < 0.01; vs. CCl_4_-treated only group, ^#^ *p* < 0.05.

In our study, the effect of FPHLP on ferroptosis in ALI model mice was evaluated via western blotting and biochemical assays. The results showed that the level of Fe^2+^ in the liver tissue of the CCl_4_-treated group was markedly elevated compared to that of the control group, while those in the FPHLP-treated groups were significantly decreased (Figure 5A). Additionally, the effect of FPHLP on ferroptosis-related protein expression, including GPX4, xCT, FTH1, and TfR1, was assessed via western blotting and the results showed that CCl_4_ markedly downregulated the expression of GPX4 and FTH1, two important proteins that chelate Fe^2+^, and upregulated the expression of xCT and TfR1, while FPHLP remarkably reversed the effect (Figure 5B,C). In all, our findings suggested that FPHLP significantly alleviated ALI via suppression of ferroptosis in mice.

### 2.6. GC-MS Analysis for FPHLP

**Figure 6:**
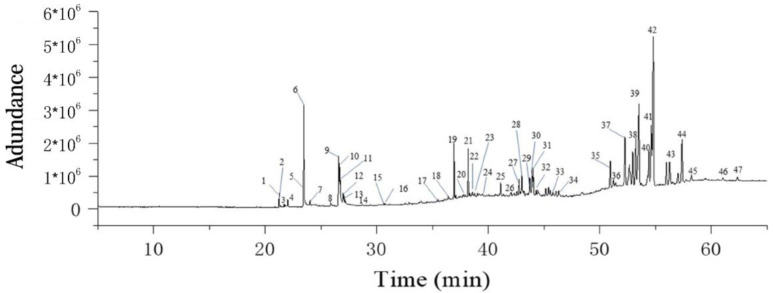
Identification of the compounds in FPHLP by GC/MS methodology: (1) neophytadiene, (2) 6,10,14-trimethyl-2-pentadecanone, (3) phytol, (4) ficusin, (5) 3,7,11,15-tetramethyl-2- hexadecen-1-ol, (6) hexadecanoic acid, (7) ethyl palmitate, (8) 5-methoxypsoralen, (9) linoleic acid, (10) oleic acid, (11) ethyl linolenate, (12) octadecanoic acid, (13) ethyl lino-leate, (14) ethyl oleate, (15) 4,8,12,16-tetramethylheptadecan-4-olide, (16) hercoyn D, (17) stigmasta-3,5-diene, (18) stigmasta-3,5-diene, (19) 1,4-benzenedicarboxylic acid,bis(2-ethylhexyl)ester, (20) Di-isononyl phthalate, (21) squalene, (22) Di-isononyl phthalate, (23) Di-isononyl phthalate, (24) dinonyl phthalate, (25) β-sitosterol acetate, (26) lupeol, (27) olean-12-en-3-yl acetate, (28) β-amyrin-3-acetate, (29) trifluoroacetate, (30) trifluoroacetate, (31) stigmasta-3,5-diene, (32) beta-amyrone, (33) psi-taraxasterol, (34) trifluoroacetate, (35) lanosta-8,24-dien-3-ol, (36) 13,27-cyclours-11-en-3-ol, (37) acetate, (38) lanosteryl acetate, (39) ole-an-12-en-3-ol, (40) taraxerol acetate, (41) lanosteryl acetate, (42) al-pha-amyrenyl acetate, (43) friedelan-3-one, (44) (3S,6aR,6bR,8aS,12S,14bR)-4,4,6a,6b,8a,11,12,14b-octamethyl-1,2,3,4,4a,5,6,6a,6b,7,8,8a,9,12,12a,12b,13,14,14a,14-bicosahydropicen-3-yl acetate, (45) dotriacontanal, (46) β-amyrenonol acetate, (47) 3.beta-acetoxy-11-oxoursan-12-ene.

The components of FPHLP were analyzed by GC-MS, and the results showed that a total of 47 major compounds were identified, including neophytadiene (peak 1), 6,10,14-trimethyl-2-pentadecanone (peak 2), phytol (peak 3), ficusin (peak 4), 3,7,11,15-tetramethyl-2-hexadecen-1-ol (peak 5), hexadecanoic acid (peak 6), ethyl palmitate (peak 7), 5-methoxypsoralen (peak 8), linoleic acid (peak 9), oleic acid (peak 10), ethyl linolenate (peak 11), octadecanoic acid (peak 12), ethyl linoleate (peak 13), ethyl oleate (peak 14), 4,8,12,16-tetramethylheptadecan-4-olide (peak 15), hercoyn D (peak 16), stigmasta-3,5-diene (peak 17), stigmasta-3,5-diene (peak 18), 1,4-benzenedicarboxylic acid,bis(2-ethylhexyl)ester (peak 19), di-isononyl phthalate (peak 20), squalene(peak 21), Di-isononyl phthalate (peak 22), di-isononyl phthalate (peak 23), dinonyl phthalate (peak 24), β-sitosterol acetate (peak 25), lupeol (peak 26), olean-12-en-3-yl acetate (peak 27), β-amyrin-3-acetate (peak 28), trifluoroacetate (peak 29), trifluoroacetate (peak 30), stigmasta-3,5-diene (peak 31), beta-amyrone (peak 32), psi-taraxasterol (peak 33), trifluoroacetate (peak 34), lanosta-8,24-dien-3-ol (peak 35), 13,27-cyclours-11-en-3-ol (peak 36), acetate (peak 37), lanosteryl acetate (peak 38), olean-12-en-3-ol (peak 39), taraxerol acetate (peak 40), lanosteryl acetate (peak 41), alpha-amyrenyl acetate (peak 42), friedelan-3-one (peak 43), (3S,6aR,6bR,8aS,12S,14bR)-4,4,6a,6b,8a,11,12,14b-octamethyl-1,2,3,4,4a,5,6,6a,6b,7,8,8a,9,12,12a,12b,13,14,14a,14-bicosahydropicen-3-yl acetate (peak 44), dotriacontanal (peak 45), β-amyrenonol acetate (peak 46), and 3beta-acetoxy-11-oxoursan-12-ene (peak 47) (Table 1 and Figure 6). The information of all compounds is shown in Table 1. It is reported that the compounds of FPHLP, such as neophytadiene [21], ethyl linoleate [22], squalene [23], phytol [24] and 5-methoxypsoralen [25], have several bioactivities, including anti-inflammation, antioxidation, and liver protection.

## 3. Material and Methods

### 3.1. Materials and Reagents

The whole plant was collected from Dajin, Kaiping, Guangdong, China. It was identified as FPH by Professor Huang Haibo from the Department of Traditional Chinese Medicine Identification of Guangzhou University of Chinese Medicine. CCl_4_ (#C11588428) was obtained from Macklin Biochemical Co., Ltd. (Shanghai, China). ALT (#C009-2-1), AST (#C010-2-1), iron (#A039-2-1), and LDH (#A020-2-2) assay kits were purchased from Nanjing Jiancheng Bioengineering Institution (Nanjing, China). GSH (#311210610), SOD (#535210610), and MDA (#417210825) ELISA kits were purchased from Tianjin Anoric Biotechnology Co., Ltd. (Tianjin, China). The IL-6 assay kit (#01/2022) was purchased from Shanghai Enzyme-linked Biotechnology Co., Ltd. (Shanghai, China). The ROS assay kit (#MM-43700M1) was purchased from Jiangsu Meimian Industrial Co., Ltd. (Yancheng, China). Primary antibodies against cleaved PARP (AF7023), Bcl2 (AF6139), GPX4 (DF6701), xCT (DF12509), FTH1 (DF6278), TfR1 (AF5343), HO-1 (AF5393), GAPDH (AF7021), and Bax (AF0120) were obtained from Affinity Biosciences Ltd. (Cincinnati, OH, USA). The primary antibody against Keap1 (D199574) was obtained from Sangon Biotech Ltd. (Shanghai, China). The primary antibody of Trx-1 (C63L6) and anti-rabbit secondary antibody (7074P2) were obtained from Cell Signaling Technology Inc. (Boston, MA, USA). The primary antibody against Nrf2 (A0674) was obtained from ABclonal Biotechnology Co., Ltd. (Wuhan, China).

### 3.2. FPHLP Preparation

FPHLP supercritical fluid extraction of FPH was performed by Nantong Huaan Supercritical Extraction Co., Ltd. (Nangtong, China) using a supercritical fluid extractor (HA220-40-11, Nangtong, China). Briefly, 2212.5 g FPH was placed into the extraction tank and carbon dioxide was pressurized by a pressurizing pump. The conditions were as follows: 45 °C extraction temperature, 55 °C separation temperature, 28 MPa extraction pressure, and 8 MPa separation pressure [26]. The extraction time was set at 3 h and the extracted essential oil was collected every 1 h. A total of 6.5 g of paste was obtained with a yield of 0.294%. According to ref. [27], the daily dosage of FPH is 100~400 g for adults, which was converted to a mice dosage of 12~50 g/kg (calculated based on the quantity of crude material). According to the yield of FPHLP (0.3%), crude drug dosages of 12 and 50 g/kg were converted into extract dosages of 36 and 150 mg/kg. We selected 50 and 100 mg/kg as the testing dosages for the animal study after a pilot study.

### 3.3. DPPH Radical Scavenging Activity Assay

A DPPH assay kit (G0128W) obtained from Suzhou Grace Biotechnology Co., Ltd. (Suzhou, China) was used to evaluate the DPPH scavenging ability of FPHLP. Briefly, 150 µL of FPHLP (40, 20, 10, 5, 2.5, and 1.25 mg/mL) was added to DPPH working solution (150 µL) and 80% methanol served as the control, according to the manufacturer’s instructions. The mixtures were co-incubated for 30 min at room temperature, then centrifuged at 5000× *g* for 5 min. A 200 µL aliquot of supernatant was transferred into a 96-well plate to measure the absorbance at 517 nm, and the DPPH scavenging rate was calculated according to the following equation:

$$\mathrm{DPPH}\;\mathrm{scavenging}\;\mathrm{rate}=\left[\frac{1-\left(\mathrm{OD}\;\mathrm{of}\;\mathrm{measurement}\;\mathrm{group}-\mathrm{OD}\;\mathrm{of}\;\mathrm{control}\;\mathrm{group}\right)}{\;\mathrm{of}\;\mathrm{blank}\;\mathrm{group}}\times 100\right]\%$$

### 3.4. Total Antioxidant Capacity (T-AOC) Analysis

According to the instructions of the T-AOC detection kit (#BC1315, Biobox, Beijing, China) [28], different concentrations of FeSO_4_ were used to establish a standard curve. The FPHLP samples were prepared in DMSO to obtain 10, 8, 6, 4, 2, and 1 mg/mL solution, and VC at 400 µg/mL was used as the positive control. The process was performed following the instructions of the T-AOC commercial kit. The blank group contained 180 µL of mixed working solution (control group) and 24 µL of distilled water. The measurement group containing 180 µL of mixed working solution, 6 µL of sample solution, and 18 µL of distilled water. The samples were co-incubated for 10 min after gentle mixing and 200 µL of the mixture was transferred to a 96-well plate to measure the absorbance of Fe^2+^-TPTZ at 592 nm. According to the standard curve, the absorbance of Fe^2+^-TPTZ was substituted to calculate the corresponding iron ion concentration. The final T-AOC was determined as follows:

T-AOC (µM) = 34 × corresponding to the concentration of iron ions.

The concentration of T-AOC was regarded as the total antioxidant capacity of FPHLP.

### 3.5. Cell Lines and Culture

HepG2 cells were purchased from the Cell Bank of Shanghai Institute of Cell Biology, Chinese Academy of Sciences (Shanghai, China). Cells were cultured in Dulbecco’s Modified Eagle Medium (DMEM) (#8120364, Gibco) containing 10% fetal bovine serum (FBS) (#2176404, Gibco) and 1% penicillin-streptomycin and maintained in an incubator under 5% CO_2_ at 37 °C. The logarithmically growing cells were used for the following experiments.

### 3.6. ROS Detection

HepG2 cells were used to evaluate the effect of FPHLP on ROS production according to ref. [29]. FPHLP was dissolved in DMSO and diluted with DMEM. Briefly, HepG2 cells were incubated with different concentrations of FPHLP (10, 8, and 6 µg/mL) for 24 h and then treated with 400 µM of H_2_O_2_ for 4 h to induce oxidative stress. The ROS level was determined using a commercial ROS assay kit (#S0033S, Beyotime Biotechnology, Shanghai, China). The cells were dyed with DCFH-DA solution and the fluorescence was detected via flow cytometry.

### 3.7. Animal Experiment Design

The animal experiment was approved by the Animal Ethics and Welfare Committee (AEWC) of the Zhongshan Hospital of Traditional Chinese Medicine. The experimental procedures and animal treatments were carried out strictly following the principle of Laboratory Animal Care and the guidelines of the AEWC of Zhongshan Hospital of Traditional Chinese Medicine (AEWC-2021026). Eight-week-old C57BL/6 male mice were provided by Zhuhai BesTest Bio-Tech Company Limited (SYXK2020-0109). All mice had access to food and water ad libitum and were kept under a 12 h light/dark cycle. The mice were randomly divided into 5 groups: control group, 0.2% CCl_4_ group, 0.2% CCl_4_ + silibinin (120 mg/kg) group, 0.2% CCl_4_ + FPHLP high dosage group (FPHLP-H, 100 mg/kg), and 0.2% CCl_4_ + FPHLP low dosage group (FPHLP-L, 50 mg/kg). The FPHLP samples and silibinin were prepared in 0.5% CMC-Na and the control group was given 0.5% CMC-Na orally from days 8 to 14. Silibinin was given orally from days 8 to 14. FPHLP was given orally from days 8 to 14, twice a day. Mice were injected with 0.2% CCl_4_ on day 13 and sacrificed after the FPHLP treatment was terminated. The experimental design is shown in Figure 2A.

### 3.8. Sample Collection

After treatment, mice were anesthetized with pentobarbital to collect blood samples and then sacrificed for dissection [30]. Blood was collected and serum was obtained by centrifugation at 4000 rpm at 4 °C for 10 min. The livers were collected and photographed. Part of the liver tissue was fixed in 4% paraformaldehyde (PFA) solution for hematoxylin and eosin (H&E) staining, and the rest was stored at −80 °C.

### 3.9. Enzyme-Linked Immunosorbent Assay (ELISA) and Biochemical Analyses

Biochemical parameters, including the concentrations of ALT and AST in serum, were determined using commercial kits according to the manufacturer’s instructions. Fresh liver tissue (50 mg) was weighed and homogenized with cold phosphate-buffered saline (0.01 M, pH 7.4, *w*/*v*, 1: 10). The samples were then centrifuged at 5000× *g* for 10 min at 4 °C, and the supernatants were collected for biochemical analysis. The levels of LDH, ROS, MDA, SOD, and GSH in liver tissues were evaluated following the manufacturer’s instructions.

### 3.10. Western Blot

Liver tissues were weighed and lysed with RIPA lysis buffer (P0013B, Beyotime Biotechnology, Shanghai, China) supplemented with 1% protease inhibitor and phosphatase inhibitor (P1046, Beyotime Biotechnology, Shanghai, China) for 30 min at 4 °C [31]. The protein samples were collected after centrifugation at 12,000 rpm for 10 min in 4 °C, and the protein concentrations were measured using a bicinchoninic acid (BCA) protein assay kit (#23225, Thermo, Rockford, USA). An equal amount of protein was loaded and separated on 8~15% sodium dodecyl sulfate-polyacrylamide gels and transferred onto polyvinylidene difluoride membranes (PVDF, Merck Millipore Ltd., IPVH00010, Darmstadt, Germany). The transferred membranes were blocked with QuickBlock^TM^ solution (P0252, Beyotime Biotechnology, Shanghai, China) for 15 min at room temperature, washed in PBST (0.1% Tween-20 in PBS), and incubated with primary antibodies of cleaved PARP, Bcl2, Bax, Keap1, HO-1, Trx-1, Nrf2, GPX4, xCT, FTH1, TfR1, and GAPDH overnight at 4 °C. After being washed with PBST 3 times, the membranes were then incubated with secondary antibodies conjugated with horseradish peroxidase (HRP) for 2 h at room temperature. The protein blots were detected using an enhanced chemiluminescence (ECL) kit (KF8003, Affinity Biosciences, Cincinnati, OH, USA). All analyses of the protein blots were performed using Image J software.

### 3.11. Hematoxylin and Eosin (H&E) Staining

Histopathological examination was performed according to the reference with minor modifications [32]. Briefly, liver tissues were fixed in 4% PFA for 24 h, dehydrated by gradient ethanol, paraffin-embedded, sectioned (~4 μm), stained with H&E, and mounted with neutral gum. The morphological changes in tissues were observed under an optical microscope (Nikon Corporation, ECLIPSE Ti2-A, Tokyo, Japan) and photos were taken (magnification, 200×).

### 3.12. Immunohistochemistry (IHC) Study

IHC analysis was performed to examine the protein expression of Nrf2 and cleaved caspase-3 in the liver tissues [33]. Briefly, the paraffin-embedded samples were cut into sections (~4 μm) and sealed with 3% H_2_O_2_ at room temperature to inactivate the enzyme, then boiled in 10 mM sodium citrate buffer (pH 6.0) for 10 min and cooled at room temperature. The sections were blocked with normal goat serum, incubated with anti-Nrf2 and anti-cleaved caspase-3 (1:200) overnight at 4 °C, and then with corresponding secondary antibodies for 1 h. The expression of Nrf2 and cleaved caspase-3 in the liver tissues was evaluated under an optical microscope and photos were taken (magnification, 200×).

### 3.13. GC/MS Analysis

To analyze the compounds, FPHLP was dissolved in chloroform for GC/MS analysis. The extraction process consisted of vortexing for 10 min, ultrasound for 30 min at room temperature, and centrifugation at 5000× *g* at 4 °C for 10 min. The characteristic components of FPHLP were analyzed using an Agilent 7890A-5975C GC/MS. The analytes were separated on a DB-5MS capillary column (60 m × 250 μm × 0.25 μm, HP-5MS, Agilent Technologies) coated with phenyl arylene polymer. The chromatographic temperature conditions used for separation were selected according to the reference, with the instrument parameters of 310 °C for the injection port temperature, 310 °C for the gas chromatography-mass spectrometer interface temperature, and N_2_ drying gas at a flow rate of 1.5 mL/min [34]. Conditions for the GC-MS analysis: 70 eV of electron energy of electron impact (EI), 35~550 of the scanning range of mass charge ratio, and 230 °C ion source temperature. MSD ChemStation software was used to process data.

### 3.14. Statistical Analysis

All data were expressed as the mean ± standard error of the mean (SEM). Statistical differences between the two groups were compared by Student’s *t*-test. Differences at *p* < 0.05 were considered statistically significant.

## 4. Discussion

A series of factors in life can lead to liver injury, such as excessive drinking, toxins, and drugs. The CCl_4_-induced liver injury model is a classical model commonly used to study liver function and liver-injury protection [35]. CCl_4_ is a potent toxin metabolized by the cytochrome P450 system that can be transformed into free radicals in the body, which disturbs the metabolism of lipids on the liver cell membrane, resulting in the production of high levels of ROS and MDA, leading to inflammation and oxidative stress [36], and eventually inducing apoptosis and necrosis of hepatocytes [37].

The treatment of liver injury mainly includes scavenging free radicals, detoxifying, reducing transaminase levels, and regulating immunity [38]. Chinese herbal medicines have been used to treat liver diseases with little side effects by suppressing inflammation and oxidative stress. For example, *Coptis chinensis* inflorescence extract exerts a hepatoprotective function by reducing ROS generation induced by CCl_4_ in HepG2 cells [39]. FPH was reported to have heat-clearing, detoxifying, and anti-inflammatory effects in the Chinese medicinal literature [31]. Our previous studies reported that the aqueous extract of FPH protected against alcohol-induced acute liver injury and alleviated colitis accompanied by secondary liver injury induced by dextran sulfate sodium (DSS) in mice [24,33]. However, no study has reported the bioactivity of FPHLP. This study showed that the low-polarity components of FPH effectively alleviated the acute liver injury in mice induced by CCl_4_.

ALT and AST leak into the serum when hepatocytes or their cell membranes are destroyed [40]. LDH, an important enzyme in glycolysis, is another key indicator of impaired liver function [41]. Inflammation dysregulation drives the liver pathology associated with acute liver injury [42], during which proinflammatory factors, such as IL-6 and LPS, promote chronic inflammation in the liver [30]. Under the pathological conditions of liver injury, excessive LPS activates innate immune cells, including Kupffer cells, leading to the release of proinflammatory cytokines such as IL-6 and TNFα, thereby reinforcing the inflammatory response [43]. Hence, inhibiting the acute inflammatory response and retarding the evolution of chronic inflammation can prevent hepatic failure from occurring. In this study, the levels of AST, ALT, LDH, IL-6, and LPS were significantly increased in the ALI mouse model induced by CCl_4_, while they were significantly reduced after FPHLP treatment, suggesting that FPHLP showed excellent efficiency against liver injury and could be regarded as a therapeutic candidate for ALI.

The activation of apoptosis is closely connected to mitochondria [44]. The activation of pro-apoptotic genes, such as Bax, results in the enhancement of mitochondrial permeabilization and release of cytochrome c, which induces caspase-3 stimulation and hepatocyte apoptosis [45]. The expression of pro-apoptotic proteins, such as Bax, cleaved caspase-3, and cleaved PARP, would be increased and that of anti-apoptotic proteins, such as Bcl2, would be decreased in impaired liver tissues [46,47], which was verified in the CCl_4_-induced ALI mouse model. In our study, FPHLP significantly reversed these effects, suggesting that FPHLP showed good efficiency in alleviating ALI via suppression of apoptosis.

Oxidative stress has become an important factor in hepatotoxicity, and increasing evidence shows that oxidative stress can promote ALI [46]. Oxidative stress can cause the imbalance of cystine/glutamate antiporter system and decrease cystine intake, thus blocking GSH synthesis [48]. GSH depletion also leads to more extensive hepatotoxicity when ROS excessively accumulates [49]. Excessive MDA covalently modifies proteins, nucleic acids, and other lipids, resulting in destruction of structural integrity or cell death [50,51]. The Keap1/Nrf2/ARE signaling pathway plays a critical role in protecting cells from endogenous and exogenous oxidative stresses [52], among which the transcription factor Nrf2 takes charge of detoxification and antioxidation and regulates GSH metabolism, while Keap1, a highly redox-sensitive member of the BTB-Kelch family, is takes responsibility for Nrf2 degradation when oxidative stress occurs [39]. Nrf2 can evade Keap1-mediated degradation, translocate to the nucleus, and activate a series of ARE-dependent genes, such as the antioxidative and cytoprotective genes HO-1 and SOD [40]. In this study, FPHLP significantly increased the levels of SOD and GSH and enhanced the expression of Nrf2 and HO-1, while inhibiting the expression of Keap1 and reducing the levels of ROS and MDA, indicating that FPHLP could alleviate ALI by suppressing oxidative stress.

Ferroptosis, a kind of non-apoptotic cell death characterized by GSH depletion and iron overloading [53], can be induced by the accumulation of redox-active iron, glutathione depletion, and lipid peroxidation [54]. Ferroptosis is involved in the occurrence and development of a series of liver diseases, including acute or chronic liver injury and liver cancer [55]. GPX4 is the key antioxidant enzyme that quenches phospholipid hydroperoxide by reducing lipid hydroperoxide to nontoxic lipid alcohol directly in the membrane. Once GSH is depleted, the Xc^−^-glutathione (GSH)-GPX4-dependent antioxidant defense system will be inactivated, leading to the accumulation of lipid hydroperoxides [53,56]. In addition, ferrous iron catalyzes and enhances the occurrence of lipid peroxidation [39]. TfR1, an identified ferroptosis marker, controls cellular iron absorption by carrying transferrin-bound iron into cells via receptor-mediated endocytosis, and then Fe^3+^ can be reduced to Fe^2+^ by STEAP3 [57]. The labile iron released from organelles upon various stresses is incorporated into enzymes, leading to production of excessive ROS that results in lipid peroxidation [40]. FTH1, a major iron storage protein harboring ferroxidase activity, also plays an important antioxidative role in maintaining the balance of the redox system [58], by taking responsibility for the oxidation of Fe^2+^ to Fe^3+^ and reducing the accumulation of Fe^2+^ [59]. As the major sites of iron utilization and master regulators of oxidative metabolism, mitochondria are the main source of ROS, which would induce apoptosis and ferroptosis of cells. Ferroptosis is also reportedly associated with severe damage of mitochondrial morphology, bioenergetics, and metabolism [60]. Our study revealed that FPHLP significantly reduced the expression of TfR1 and accumulation of Fe^2+^ and increased the expression of GPX4 and FTH1, suggesting that FPHLP effectively suppressed ferroptosis when treating ALI in mice. Furthermore, ferroptosis can also propagate lipid peroxidation chain reactions, leading to hepatocyte destruction and apoptotic death [61]. Injured hepatocytes not only jeopardize liver function, but they also activate Kupffer cells, resulting in proinflammatory and fibrogenic responses and the vicious cycle of liver damage [62].

Altogether, our study indicated that FPHLP significantly protected the liver from injury in mice with CCl_4_-induced ALI by suppressing oxidative stress, inflammation, ferroptosis, and apoptosis, suggesting that FPHLP may be regarded as a potential hepatoprotective drug. However, the key active ingredient and target of FPHLP remain undefined, which will be investigated in the follow-up study.

## 5. Conclusions

**Figure 7:**
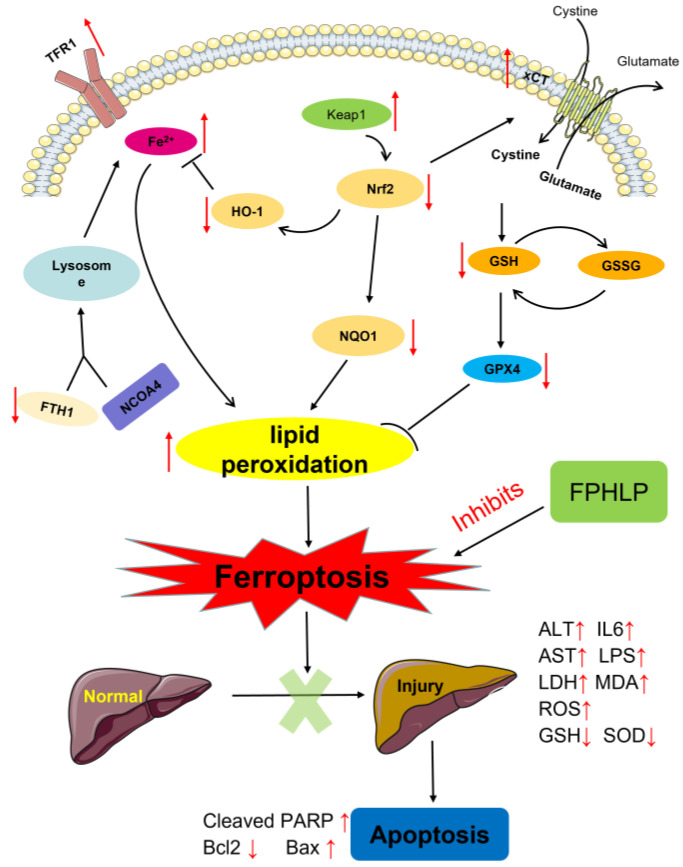
The speculated mechanism of FPHLP against ALI in mice induced by CCl_4_. Note: ↑ increases; ↓ decreases.

In conclusion, this is the first study to explore the suppression of ALI by FPHLP as well as the underlying mechanism. The in vitro data showed that FPHLP efficiently suppressed oxidative stress, and the in vivo study indicated that FPHLP showed excellent inhibition against CCl4-induced ALI in a mouse model. FPHLP improved liver function by reducing ALT and ALT levels in ALI model mice and improved the antioxidant capacity by increasing the levels of antioxidant enzymes, such as SOD and GSH. In addition, FPHLP reduced the accumulation of Fe^2+^ by enhancing the iron transport mechanism (Figure 7). Our study provides experimental evidence for the therapeutic function of FPHLP against ALI, suggesting that FPHLP might serve as a novel candidate for the treatment of liver injury.

## Figures and Tables

**Table 1:** Identified chemical constituents and their % content in FPHLP by GC/MS.

Peak No.	Identified Compound Name	Molecular Formula	Molecular Weight
1	Neophytadiene	C_20_H_38_	278.3
2	6,10,14-trimethyl-2-Pentadecanone	C_18_H_36_O	268.28
3	Phytol	C_20_H_40_O	296.53
4	Ficusin	C_11_H_6_O_3_	186.16
5	3,7,11,15-Tetramethyl-2-hexadecen-1-ol	C_20_H_40_O	296.3
6	Hexadecanoic acid	C_16_H_32_O_2_	256.24
7	Ethyl palmitate	C_18_H_36_O_2_	284.27
8	5-Methoxypsoralen	C_12_H_8_O_4_	216.04
9	Linoleic acid	C_18_H_32_O_2_	280.24
10	Oleic acid	C18H34O2	282.26
11	Ethyl linolenate	C_20_H_34_O_2_	306.26
12	Octadecanoic acid	C_18_H_36_O_2_	284.27
13	Ethyl linoleate	C_20_H_36_O_2_	308.27
14	Ethyl oleate	C_20_H_38_O_2_	310.29
15	4,8,12,16-Tetramethylheptadecan-4-olide	C_21_H_40_O_2_	324.3
16	Hercoyn D	C_21_H_30_O_2_	314.23
17	Stigmasta-3,5-diene	C_29_H_48_	396.38
18	Stigmasta-3,5-diene	C_29_H_48_	396.38
19	1,4-Benzenedicarboxylic acid, bis(2-ethylhexyl) ester	C_24_H_38_O_4_	390.56
20	Di-isononyl phthlate	C_26_H_42_O_4_	418.31
21	Squalene	C_30_H_50_	410.39
22	Di-isononyl phthlate	C_26_H_42_O_4_	418.31
23	Di-isononyl phthlate	C_26_H_42_O_4_	418.31
24	Dinonyl phthalate	C_26_H_42_O_4_	418.31
25	β-Sitosterol acetate	C_31_H_52_O_2_	456.39
26	Lupeol	C_32_H_49_F_3_O_2_	522.37
27	Olean-12-en-3-yl acetate	C_32_H_52_O_2_	468.39
28	β-Amyrin 3-acetate	C_32_H_52_O_2_	468.39
29	Trifluoroacetate	C_32_H_49_F_3_O_2_	522.37
30	Trifluoroacetate	C_32_H_49_F_3_O_2_	522.37
31	Stigmasta-3,5-diene	C_29_H_48_	396.37
32	beta.-Amyrone	C_30_H_48_O	424.7
33	Psi-taraxasterol	C_30_H_50_O	426.72
34	Trifluoroacetate	C_32_H_49_F_3_O_2_	522.37
35	Lanosta-8,24-dien-3-ol,	C_32_H_52_O_2_	468.75
36	13,27-Cyclours-11-en-3-ol, acetate	C_32_H_50_O_2_	466.38
37	Lanosta-8,24-dien-3-ol,	C_32_H_50_O_2_	466.38
38	Lanosteryl acetate	C_32_H_52_O_2_	468.75
39	Olean-12-en-3-ol	C_32_H_52_O_2_	468.75
40	Taraxerol acetate	C_32_H_52_O_2_	468.75
41	Lanosteryl acetate	C_32_H_52_O_2_	468.75
42	alpha-Amyrenyl acetate	C_32_H_52_O_2_	468.75
43	Friedelan-3-one	C_30_H_50_O	426.72
44	(3S,6aR,6bR,8aS,12S,14bR)-4,4,6a,6b,8a,11,12,14b-octamethyl-1,2,3,4,4a,5,6,6a,6b,7,8,8a,9,12,12a,12b,13,14,14a,14b-icosahydropicen-3-yl acetate	C_32_H_52_O_2_	468.39
45	Dotriacontanal	C_32_H_64_O	464.49
46	β-Amyrenonol acetate	C_32_H_50_O_3_	482.74
47	3.beta-Acetoxy-11-oxoursan-12-ene	C_32_H_50_O_3_	482.74

## Data Availability

The data presented in this study are available upon reasonable request from the corresponding author.

## Abbreviations

AEWC	animal ethics and welfare committee
ALI	acute liver injury
ALT	alanine aminotransferase
AST	aspartate aminotransferase
Bax	Bcl2 associated X protein
BCA	bicinchoninic acid
Bcl2	B-cell lymphoma-2
Cleaved PARP	cleaved poly ADP-ribose polymerase
CYP450	cytochrome P450
DMEM	Dulbecco’s modified eagle medium
DMSO	dimethyl Sulfoxide
DPPH	2,2-diphenyl-1-picrylhydrazil
ECL	enhanced chemiluminescence
EI	electron impact
ELISA	enzyme-linked immunosorbent assay
FBS	fetal bovine serum
Fe^2+-^TPTZ	ferric-tripyridyl-triazine
FPH	*Ficus pandurata* Hance
FPHLP	low-polarity ingredients of *Ficus pandurata* Hance
FRAP	ferric reducing antioxidant power
FTH1	ferritin heavy chain
GAPDH	glyceraldehyde-3-phosphate dehydrogenase
Gas	chromatography-mass spectrometry
GC-MS	gas chromatography-mass spectrometry
GPX4	glutathione peroxidase 4
GSH	glutathione
H&E	Hematoxylin & Eosin
HO-1	oxygenase-1
IBD	inflammatory bowel disease
IHC	immunohistochemistry
IL-6	interleukin- 6
Keap1	kelch-like ECH-associated protein 1
LDH	lactate dehydrogenase
LPS	lipopolysaccharide
MDA	malondialdehyde
NAC	N-acetyl-L-cysteine
Nrf2	nuclear factor erythroid 2-related factor 2
OD	optical density
PBS	phosphate buffer saline
PBST	phosphate buffer saline with Tween 20
PFA	paraformaldehyde
PVDF	polyvinylidene difluoride membranes
ROS	reactive oxygen species
SOD	superoxide dismutase
STEAP3	six-transmembrane epithelial antigen of prostate 3
T-AOC	total antioxidant capacity
TfR1	transferrin receptor 1
Trx	thioredoxin
VC	Vitamin C
xCT/SLC7A11	cystine/glutamate antiporter solute carrier family 7 member 11

## References

[1] Shen T., Liu Y., Shang J., Xie Q., Li J., Yan M., Xu J., Niu J., Liu J., Watkins P.B. (2019). Incidence and Etiology of Drug-Induced Liver Injury in Mainland China. Gastroenterology.

[2] Williams R., Alexander G., Armstrong I., Baker A., Bhala N., Camps-Walsh G., Cramp M.E., de Lusignan S., Day N., Dhawan A. (2018). Disease burden and costs from excess alcohol consumption, obesity, and viral hepatitis: Fourth report of the Lancet Standing Commission on Liver Disease in the UK. Lancet.

[3] Kim J.B., Sohn J.H., Lee H.L., Kim J.P., Han D.S., Hahm J.S., Lee D.H., Kee C.S. (2004). Clinical characteristics of acute toxic liver injury. Korean J. Hepatol..

[4] Ibanez L., Perez E., Vidal X., Laporte J.R., Grup d’Estudi Multicènteric d’Hepatotoxicitat Aguda de Barcelona (GEMHAB) (2002). Prospective surveillance of acute serious liver disease unrelated to infectious, obstructive, or metabolic diseases: Epidemiological and clinical features, and exposure to drugs. J. Hepatol..

[5] Krawitz S., Lingiah V., Pyrsopoulos N.T. (2018). Acute Liver Failure: Mechanisms of Disease and Multisystemic Involvement. Clin. Liver Dis..

[6] Yang Y.M., Cho Y.E., Hwang S. (2022). Crosstalk between Oxidative Stress and Inflammatory Liver Injury in the Pathogenesis of Alcoholic Liver Disease. Int. J. Mol. Sci..

[7] Pan X., Zhou J., Chen Y., Xie X., Rao C., Liang J., Zhang Y., Peng C. (2020). Classification, hepatotoxic mechanisms, and targets of the risk ingredients in traditional Chinese medicine-induced liver injury. Toxicol. Lett..

[8] Yi J., Wu S., Tan S., Qin Y., Wang X., Jiang J., Liu H., Wu B. (2021). Berberine alleviates liver fibrosis through inducing ferrous redox to activate ROS-mediated hepatic stellate cells ferroptosis. Cell Death Discov..

[9] Wu J., Wang Y., Jiang R., Xue R., Yin X., Wu M., Meng Q. (2021). Ferroptosis in liver disease: New insights into disease mechanisms. Cell Death Discov..

[10] Thomas C.E., Morehouse L.A., Aust S.D. (1985). Ferritin and superoxide-dependent lipid peroxidation. J. Biol. Chem..

[11] Dixon S.J., Lemberg K.M., Lamprecht M.R., Skouta R., Zaitsev E.M., Gleason C.E., Patel D.N., Bauer A.J., Cantley A.M., Yang W.S. (2012). Ferroptosis: An iron-dependent form of nonapoptotic cell death. Cell.

[12] Brok J., Buckley N., Gluud C. (2002). Interventions for paracetamol (acetaminophen) overdoses. Cochrane Database Syst. Rev..

[13] Wu J., Lu A.D., Zhang L.P., Zuo Y.X., Jia Y.P. (2019). Study of clinical outcome and prognosis in pediatric core binding factor-acute myeloid leukemia. Zhonghua Xue Ye Xue Za Zhi.

[14] Li M., Luo Q., Tao Y., Sun X., Liu C. (2021). Pharmacotherapies for Drug-Induced Liver Injury: A Current Literature Review. Front. Pharmacol..

[15] Stine J.G., Lewis J.H. (2016). Current and future directions in the treatment and prevention of drug-induced liver injury: A systematic review. Expert Rev. Gastroenterol. Hepatol..

[16] Oray M., Abu Samra K., Ebrahimiadib N., Meese H., Foster C.S. (2016). Long-term side effects of glucocorticoids. Expert Opin. Drug Saf..

[17] Garcia-Cortes M., Ortega-Alonso A., Andrade R.J., Spanish Group for the Study of Drug-Induced Liver D. (2022). Safety of treating acute liver injury and failure. Expert Opin. Drug Saf..

[18] Chalasani N.P., Maddur H., Russo M.W., Wong R.J., Reddy K.R., Practice Parameters Committee of the American College of Gastroenterology (2021). ACG Clinical Guideline: Diagnosis and Management of Idiosyncratic Drug-Induced Liver Injury. Am. J. Gastroenterol..

[19] Singh D., Cho W.C., Upadhyay G. (2015). Drug-Induced Liver Toxicity and Prevention by Herbal Antioxidants: An Overview. Front. Physiol..

[20] Zhang Y., Luo J., Zeng F. (2021). Volatile composition analysis of tree peony (Paeonia section Moutan DC.) seed oil and the effect of oxidation during storage. J. Food Sci..

[21] Dai W., Chen C., Feng H., Li G., Peng W., Liu X., Yang J., Hu X. (2021). Protection of Ficus pandurata Hance against acute alcohol-induced liver damage in mice via suppressing oxidative stress, inflammation, and apoptosis. J. Ethnopharmacol..

[22] Liu Y., Huang Z.Z., Min L., Li Z.F., Chen K. (2021). The BRD4 inhibitor JQ1 protects against chronic obstructive pulmonary disease in mice by suppressing NF-kappaB activation. Histol. Histopathol..

[23] Tan J., Li P., Xue H., Li Q. (2020). Cyanidin-3-glucoside prevents hydrogen peroxide (H(2)O(2))-induced oxidative damage in HepG2 cells. Biotechnol. Lett..

[24] Zatroch K.K., Knight C.G., Reimer J.N., Pang D.S. (2017). Refinement of intraperitoneal injection of sodium pentobarbital for euthanasia in laboratory rats (*Rattus norvegicus*). BMC Vet. Res..

[25] Dai W., Zhan X., Peng W., Liu X., Peng W., Mei Q., Hu X. (2021). Ficus pandurata Hance Inhibits Ulcerative Colitis and Colitis-Associated Secondary Liver Damage of Mice by Enhancing Antioxidation Activity. Oxid. Med. Cell. Longev..

[26] Zhan X., Peng W., Wang Z., Liu X., Dai W., Mei Q., Hu X. (2022). Polysaccharides from Garlic Protect against Liver Injury in DSS-Induced Inflammatory Bowel Disease of Mice via Suppressing Pyroptosis and Oxidative Damage. Oxid Med. Cell. Longev..

[27] Chen Y., Zhang P., Chen W., Chen G. (2020). Ferroptosis mediated DSS-induced ulcerative colitis associated with Nrf2/HO-1 signaling pathway. Immunol. Lett..

[28] Yu X., Zhang H., Wang J., Wang J., Wang Z., Li J. (2022). Phytochemical Compositions and Antioxidant Activities of Essential Oils Extracted from the Flowers of Paeonia delavayi Using Supercritical Carbon Dioxide Fluid. Molecules.

[29] Wang K. (2014). Molecular mechanisms of hepatic apoptosis. Cell Death Dis..

[30] Bhardwaj M., Sali V.K., Mani S., Vasanthi H.R. (2020). Neophytadiene from Turbinaria ornata Suppresses LPS-Induced Inflammatory Response in RAW 264.7 Macrophages and Sprague Dawley Rats. Inflammation.

[31] Park S.Y., Seetharaman R., Ko M.J., Kim D.Y., Kim T.H., Yoon M.K., Kwak J.H., Lee S.J., Bae Y.S., Choi Y.W. (2014). Ethyl linoleate from garlic attenuates lipopolysaccharide-induced pro-inflammatory cytokine production by inducing heme oxygenase-1 in RAW264.7 cells. Int. Immunopharmacol..

[32] Chen Y.P., Gu Y.F., Zhao H.R., Zhou Y.M. (2021). Dietary squalene supplementation alleviates diquat-induced oxidative stress and liver damage of broiler chickens. Poult. Sci..

[33] Kim E.N., Trang N.M., Kang H., Kim K.H., Jeong G.S. (2022). Phytol Suppresses Osteoclast Differentiation and Oxidative Stress through Nrf2/HO-1 Regulation in RANKL-Induced RAW264.7 Cells. Cells.

[34] Liang Y., Xie L., Liu K., Cao Y., Dai X., Wang X., Lu J., Zhang X., Li X. (2021). Bergapten: A review of its pharmacology, pharmacokinetics, and toxicity. Phytother. Res..

[35] Xu G., Han X., Yuan G., An L., Du P. (2017). Screening for the protective effect target of deproteinized extract of calf blood and its mechanisms in mice with CCl4-induced acute liver injury. PLoS ONE.

[36] Unsal V., Cicek M., Sabancilar I. (2021). Toxicity of carbon tetrachloride, free radicals and role of antioxidants. Rev. Environ. Health.

[37] Kawakami K., Moritani C., Hatanaka T., Suzaki E., Tsuboi S. (2020). Hepatoprotective Activity of Yellow Chinese Chive against Acetaminophen-Induced Acute Liver Injury via Nrf2 Signaling Pathway. J. Nutr. Sci. Vitaminol..

[38] Muniz Santana Bastos E., Bispo da Silva A., Cerqueira Coelho P.L., Pereira Borges J.M., Amaral da Silva V.D., Moreau da Cunha V.H., Costa S.L. (2021). Anti-inflammatory activity of Jatropha curcas L. in brain glial cells primary cultures. J. Ethnopharmacol..

[39] Lam P., Cheung F., Tan H.Y., Wang N., Yuen M.F., Feng Y. (2016). Hepatoprotective Effects of Chinese Medicinal Herbs: A Focus on Anti-Inflammatory and Anti-Oxidative Activities. Int. J. Mol. Sci..

[40] Kew M.C. (2000). Serum aminotransferase concentration as evidence of hepatocellular damage. Lancet.

[41] Puri B.K., Kingston M.C., Monro J.A. (2019). Fructose-associated hepatotoxicity indexed by the lactate dehydrogenase isoenzyme LDH-5. Med. Hypotheses.

[42] Robinson M.W., Harmon C., O’Farrelly C. (2016). Liver immunology and its role in inflammation and homeostasis. Cell Mol. Immunol.

[43] Thakur V., McMullen M.R., Pritchard M.T., Nagy L.E. (2007). Regulation of macrophage activation in alcoholic liver disease. J. Gastroenterol. Hepatol..

[44] Ahmadian E., Eftekhari A., Kavetskyy T., Khosroushahi A.Y., Turksoy V.A., Khalilov R. (2020). Effects of quercetin loaded nanostructured lipid carriers on the paraquat-induced toxicity in human lymphocytes. Pestic. Biochem. Physiol..

[45] Ahmadian E., Khosroushahi A.Y., Eftekhari A., Farajnia S., Babaei H., Eghbal M.A. (2018). Novel angiotensin receptor blocker, azilsartan induces oxidative stress and NFkB-mediated apoptosis in hepatocellular carcinoma cell line HepG2. Biomed. Pharmacother..

[46] Ma J.Q., Ding J., Zhang L., Liu C.M. (2014). Hepatoprotective properties of sesamin against CCl4 induced oxidative stress-mediated apoptosis in mice via JNK pathway. Food Chem. Toxicol..

[47] Volarevic V., Misirkic M., Vucicevic L., Paunovic V., Simovic Markovic B., Stojanovic M., Milovanovic M., Jakovljevic V., Micic D., Arsenijevic N. (2015). Metformin aggravates immune-mediated liver injury in mice. Arch. Toxicol..

[48] Lewerenz J., Hewett S.J., Huang Y., Lambros M., Gout P.W., Kalivas P.W., Massie A., Smolders I., Methner A., Pergande M. (2013). The cystine/glutamate antiporter system x(c)(-) in health and disease: From molecular mechanisms to novel therapeutic opportunities. Antioxid. Redox Signal..

[49] Eftekhari A., Ahmadian E., Azarmi Y., Parvizpur A., Hamishehkar H., Eghbal M.A. (2016). In vitro/vivo studies towards mechanisms of risperidone-induced oxidative stress and the protective role of coenzyme Q10 and N-acetylcysteine. Toxicol. Mech. Methods.

[50] Del Rio D., Stewart A.J., Pellegrini N. (2005). A review of recent studies on malondialdehyde as toxic molecule and biological marker of oxidative stress. Nutr. Metab. Cardiovasc. Dis..

[51] Patlevic P., Vaskova J., Svorc P., Vasko L., Svorc P. (2016). Reactive oxygen species and antioxidant defense in human gastrointestinal diseases. Integr. Med. Res..

[52] Rosowsky A., Wright J.E., Holden S.A., Waxman D.J. (1990). Influence of lipophilicity and carboxyl group content on the rate of hydroxylation of methotrexate derivatives by aldehyde oxidase. Biochem. Pharmacol.

[53] Yang W.S., Stockwell B.R. (2016). Ferroptosis: Death by Lipid Peroxidation. Trends Cell Biol..

[54] Bertrand R.L. (2017). Iron accumulation, glutathione depletion, and lipid peroxidation must occur simultaneously during ferroptosis and are mutually amplifying events. Med. Hypotheses.

[55] Capelletti M.M., Manceau H., Puy H., Peoc’h K. (2020). Ferroptosis in Liver Diseases: An Overview. Int. J. Mol. Sci..

[56] Chen X., Li J., Kang R., Klionsky D.J., Tang D. (2021). Ferroptosis: Machinery and regulation. Autophagy.

[57] Zheng J., Conrad M. (2020). The Metabolic Underpinnings of Ferroptosis. Cell Metab..

[58] Wei L., Zuo Z., Yang Z., Yin H., Yang Y., Fang J., Cui H., Du Z., Ouyang P., Chen X. (2022). Mitochondria damage and ferroptosis involved in Ni-induced hepatotoxicity in mice. Toxicology.

[59] Fang Y., Chen X., Tan Q., Zhou H., Xu J., Gu Q. (2021). Inhibiting Ferroptosis through Disrupting the NCOA4-FTH1 Interaction: A New Mechanism of Action. ACS Cent. Sci..

[60] Battaglia A.M., Chirillo R., Aversa I., Sacco A., Costanzo F., Biamonte F. (2020). Ferroptosis and Cancer: Mitochondria Meet the “Iron Maiden” Cell Death. Cells.

[61] Su L.J., Zhang J.H., Gomez H., Murugan R., Hong X., Xu D., Jiang F., Peng Z.Y. (2019). Reactive Oxygen Species-Induced Lipid Peroxidation in Apoptosis, Autophagy, and Ferroptosis. Oxid. Med. Cell. Longev..

[62] Czaja A.J. (2014). Hepatic inflammation and progressive liver fibrosis in chronic liver disease. World J. Gastroenterol..

